# Transmission of *Armillifer armillatus* Ova at Snake Farm, The Gambia, West Africa

**DOI:** 10.3201/eid1702.101118

**Published:** 2011-02

**Authors:** Dennis Tappe, Michael Meyer, Anett Oesterlein, Assan Jaye, Matthias Frosch, Christoph Schoen, Nikola Pantchev

**Affiliations:** Author affiliations: University of Würzburg, Würzburg, Germany (D. Tappe, A. Oesterlein, M. Frosch, C. Schoen);; Touray and Meyer Veterinary Clinic, Bijilo, The Gambia (M. Meyer);; Medical Research Council Laboratories, Fajara, The Gambia (A. Jaye);; Veterinary Medical Laboratory, Ludwigsburg, Germany (N. Pantchev)

**Keywords:** Parasites, zoonoses, agriculture, occupational exposures, pentastomiasis, Armillifer armillatus, pentastomes, The Gambia, dispatch

## Abstract

Visceral pentastomiasis caused by *Armillifer armillatus* larvae was diagnosed in 2 dogs in The Gambia. Parasites were subjected to PCR; phylogenetic analysis confirmed relatedness with branchiurans/crustaceans. Our investigation highlights transmission of infective *A. armillatus* ova to dogs and, by serologic evidence, also to 1 human, demonstrating a public health concern.

Pentastomes are an unusual group of vermiform parasites that infect humans and animals. Phylogenetically, these parasites represent modified crustaceans probably related to maxillopoda/branchiurans (*1*). Most documented human infections are caused by members of the species *Armillifer armillatus*, which cause visceral pentastomiasis in West and Central Africa (*2**–**4*). An increasing number of infections are reported from these regions (*5**–**7*). Close contact with snake excretions, such as in python tribal totemism in Africa (*5*) and tropical snake farming (*2*), as well as consumption of undercooked contaminated snake meat (*8*), likely plays a major role in transmission of pentastome ova to humans.

## The Study

**Figure 1:**
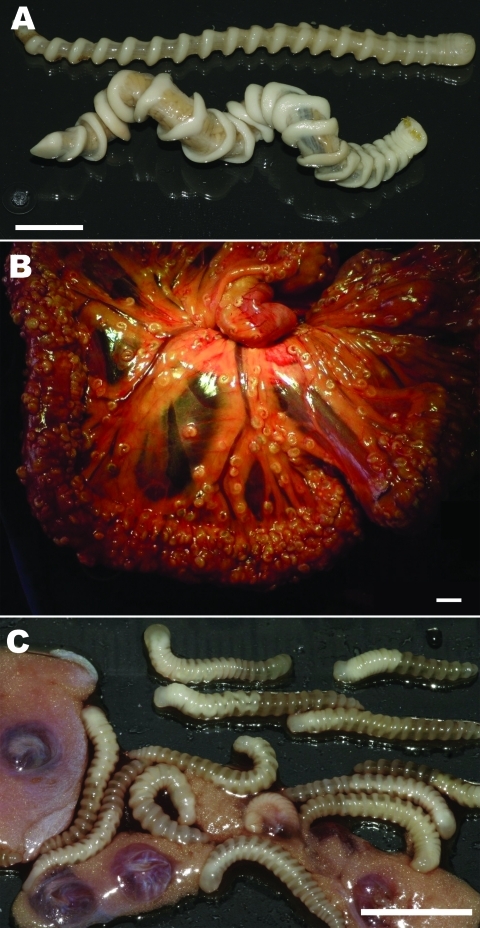
Adult and larval *Armillifer armillatus* parasites. A) Ventral view of 2 adult *A. armillatus* parasites recovered from the lungs and trachea of a deceased rock python; a gravid female (bottom) and a pre-adult female (top) are shown. The parasites showed 20 and 18 marked body rings, and had a length of 10 cm and 9 cm and a body width of 5–8 mm and 3–5 mm, respectively. B) Heavily parasitized omentum of a female stray dog, showing typical encapsulated C-shaped larval stages of *A. armillatus* parasites. C) Larvae from the omentum. The larvae had a length of 18–19 mm and a body width of 2 mm and showed 20–22 rings. Scale bars = 1 cm.

In May 2009, a 7-year-old female dog was admitted to a veterinary clinic in Bijilo, The Gambia, for elective ovariohysterectomy. The owner of the dog, a snake farm operator, reported late abortions during several pregnancies of the animal. The dog had been kept on the farm premises, where adult snakes (African rock pythons, *Python sebae*) had died several months before of infection with adult *A. armillatus* pentastomes (Figure 1, panel A). During the dog’s surgery, hundreds of pentastomid larvae were seen on the enteral serosa, bladder, uterus, and in the omentum (Figure 1, panels B, C). In April 2010, a male stray dog, 6 months of age, was admitted to the veterinary clinic for elective neutering. Coiled pentastomid larvae were found in the vaginal processes of the testes during surgery. Adult and larval parasite specimens were preserved in 100% ethanol for further parasitologic, histologic, and molecular examinations.

To investigate the extent of infection and determine whether transmission to humans on the snake farm grounds had occurred, we collected serum specimens from the 46-year-old male Caucasian snake farm owner, his 28-year-old wife, his 3 children, and the infected female dog. All human patients were asymptomatic, and informed consent was obtained. Serum samples were transferred to the Institute of Hygiene and Microbiology (University of Würzburg, Würzburg, Germany) for analysis by ELISA and Western blot based on larval parasite antigens.

**Figure 2:**
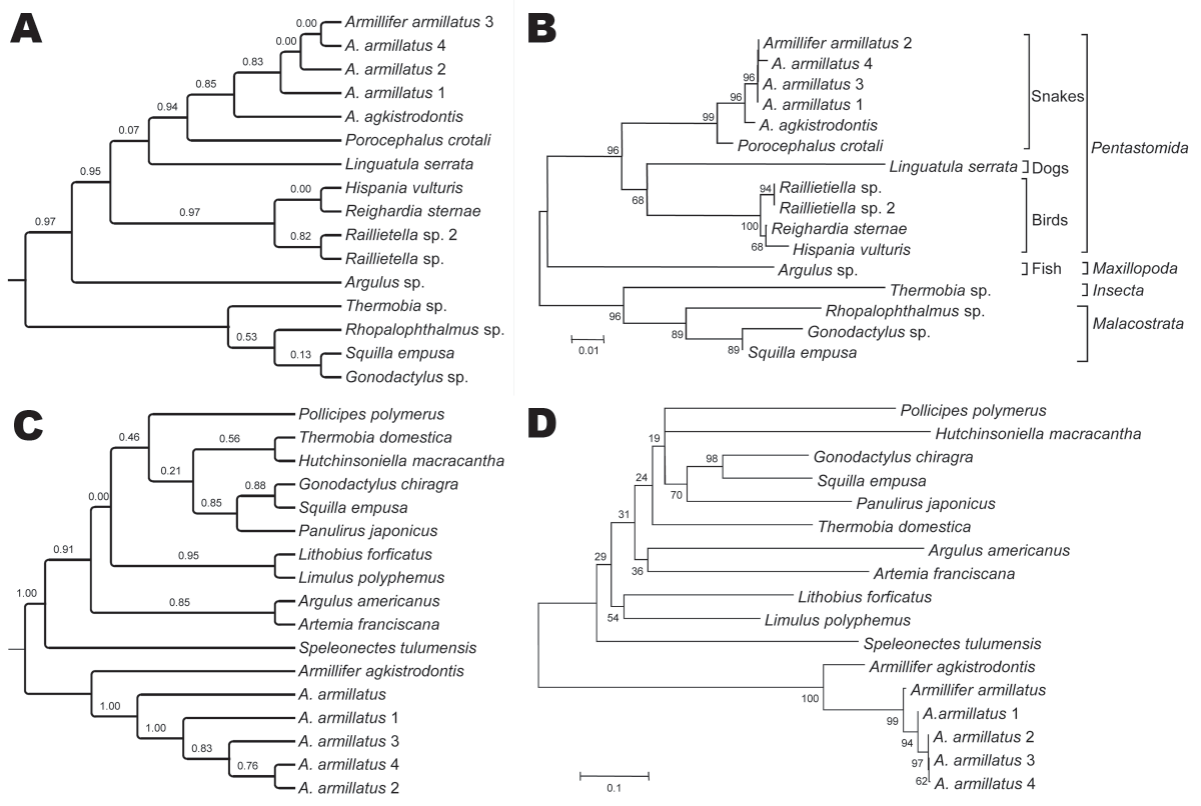
Molecular phylogeny of 4 *Armillifer* *amillatus* specimens based on partial 18S rRNA and partial cytochrome c oxidase (*cox*) gene sequences. Panels A and C show cladograms based on maximum-likelihood (ML); panels B and D show minimum-evolution (ME). In the cladograms, the approximate likelihood ratios are given next to the branches to indicate the statistical support for the respective branches. In panels B and D, the percentage of replicate trees in which the associated taxa clustered together in the bootstrap test (1,000 replicates) is shown next to the branches, and the evolutionary distances are given as scale bars as the number of base substitutions per site. The phylogram of the partial 18S rRNA sequences (B) depicts respective host species for the parasitic Pentastomida. The bird parasites *Raillietiella* sp., *Reighardia sternae*, and *Hispania vulturis* belong to the order Cephalobaenida, whereas snake parasites *Armillifer* spp. and *Porocephalus* spp. and dog parasites *Linguatula* spp. belong to the order Porocephalida. Note that not all species have entries for 18S rRNA and *cox* genes in GenBank. GenBank accession numbers of the species depicted can be found in the Table (Table). MUSCLE (www.ebi.ac.uk/Tools/muscle/index.html) and RevTrans 1.4 (www.cbs.dtu.dk/services/RevTrans/) were used for the alignment of partial 18S rDNA and *cox* sequences, respectively. Poorly aligned regions were removed with Gblocks. MODELTEST (darwin.uvigo.es/software/modeltest.html) was used for the selection of the substitution models. ML analyses of the 18S rDNA sequences were performed with PhyML 3.0 (http://atgc.lirmm.fr/phyml/) under the TN93 + I + Γ substitution model (6 rate classes and NNI algorithm for tree searching). ML analyses of the *cox* sequences were performed under the generalized time reversible + I + Γ substitution model. Approximate likelihood ratios were used to estimate the branch supports of the inferred ML phylogeny, which was visualized with TreeGraph2. MEGA4 (www.megasoftware.net) was used for ME analysis of the 18S rDNA sequences under the TN93 + I + Γ substitution model, and with maximum composite likelihood for *cox* sequences. The close neighbor interchange algorithm was used for tree searching, with pair-wise deletion of sequence gaps and considering differences in the substitution pattern among lineages.

DNA of the pentastome specimens (2 adults from the snakes, 1 larva from each dog) was extracted by using the QIAGEN Tissue Kit (QIAGEN, Hilden, Germany) and subjected to 18S rRNA and cytochrome c oxidase (*cox*) gene PCR with primers Pent629F (5′-CGGTTAAAAAGCTCGTAGTTGG-3′) and Pent629R (5′-GGCATCGTTTATGGTTAGAACTAGGG-3′ [*9*]) and primers Cox1-F (5′-CTGCGACAATGACTATTTTCAAC-3′) and Cox1-R (5′-ATATGGGAAGTTCTGAGTAGG-3′ [*10*]). After sequencing and BLAST (*www.ncbi.nlm.nih.gov/blast)* analysis of the partial 18S rRNA gene amplicons, the sequences showed high homology with *A. agkistrodontis* and *Porocephalus crotali* parasites*.* However, no *A. armillatus* 18S rRNA gene entry existed in GenBank; *cox* gene sequences showed high homology with *A. armillatus*, followed by *A. agkistrodontis*. The 4 amplified partial 18S rRNA gene sequences were 100% identical, as expected for the same species, but heterogenicity was seen in the *cox* sequences (99.7% identity). When phylogenetic trees were constructed, the nearest neighbor of *A. armillatus* was *A. agkistrodontis* in both models for both genes, followed by *P. crotali* and *Linguatula serrata* (18S rRNA only as no *cox* entry existed). All pentastomes analyzed clustered together and formed their own branch, with *Argulus* sp. being the nearest maxillopodan/branchiuran neighbor in both models by using 18S rRNA sequence data, and *Speleonectes tulumensis* (Remipedia) crustaceans when *cox* sequences were used (Figure 2). The 18S sequence of *A. armillatus* was submitted to GenBank (accession no. HM756289).

**Table:** Sequences used for phylogenetic inferences of Armillifer armillatus, The Gambia, 2009–2010

Species and/or genera used	GenBank accession no.
18S rRNA gene PCR	
*A. armillatus* (samples 1–4)	HM756289 (this study)
* A. agkistrodontis*	FJ607339.1
* Porocephalus crotali*	M29931.1
* Linguatula serrata*	FJ528908.1
*Raillietiella* sp.	AY744887.1, EU370434.1
* Reighardia sternae*	AY304521.1
* Hispania vulturis*	AY304520.1
*Argulus* sp.	DQ531766.1
*Thermobia* sp.	AY338726.1
*Rhopalophthalmus* sp.	AM422488.1
*Gonodactylus* sp.	L81947.1
* Squilla empusa*	L81946.1
Cytochrome oxidase gene PCR	
* A. armillatus*	AY456186.1
* A. agkistrodontis*	FJ607340.1
* Speleonectes tulumensis*	AY456190.1
* Argulus americanus*	AY456187.1
* Artemia franciscana*	NC_001620.1
* Lithobius forficatus*	AF309492.1
* Limulus polyphemus*	NC_003057.1
* Thermobia domestica*	AY639935.1
* Panulirus japonicus*	NC_004251.1
* Gonodactylus chiragra*	NC_007442.1
* Pollicipes polymerus*	NC_005936.1
* Hutchinsonella macracantha*	AY456189.1
* S. empusa*	NC_007444.1

The crude parasite antigen ELISA was set up in a similar manner to an in-house *Echinococcus multilocularis* ELISA (*11*) by using larvae from the canine omentum. Because no serum specimens from persons with proven *Armillifer* spp. infections were available as positive controls, a stored serum sample was used from a patient with a histologically confirmed *L. serrata* infection (*12*). Ten serum samples from healthy German blood donors served as negative controls, and a standardized threshold index of 1.0 was calculated (*11*). In addition, the serum of the infected female dog was tested, as well as 10 serum samples from uninfected dogs from Germany. The crude larval antigen was also used in a Western blot (2 µg/slot), and all serum samples from The Gambia and the serum sample from the *Linguatula* spp.–infected patient were analyzed. Of the serum samples tested, only the serum sample from the snake farm owner was positive for pentastomiasis in the ELISA, index 1.2. All other persons had indices below the threshold index (0.71–0.30). The control serum sample from the patient with linguatuliasis exhibited an index of 1.3.

When tested by Western blot, the serum of the farm owner demonstrated a banding pattern similar to the *L. serrata*–positive control serum. Both serum samples exhibited 97-kDa and 37-kDa bands, and the serum from the patient with linguatuliasis had an additional 50-kDa band (not shown). All human serum samples were negative for various helminthic diseases. When dog serum samples were tested, only the sample from The Gambia showed a positive reaction in the ELISA, with an index of 1.0.

## Conclusions

Pentastomiasis is a parasitic zoonosis with an increasing number of recognized human infections in West Africa. Our investigation highlights the local transmission of infective *A. armillatus* ova to dogs and, by serologic evidence, also to 1 human, and thus demonstrates a public health concern. Possibly because of their eating habits (e.g., consumption of dead snakes), dogs seem to be at high risk and could function as sentinel animals. In this study, we set up serologic assays for pentastomiasis based on raw larval *A. armillatus* antigens and screened the farm workers for past infection. The infection of 1 person, the snake farm owner, could be demonstrated by ELISA and Western blot for human serum samples. In 1982, an indirect immunofluorescence assay based on *A. armillatus* larvae was used for a survey in the Ivory Coast; results indicated a low seroprevalence (*13*).

In most human cases, pentastomiasis is asymptomatic and is an incidental finding during surgery or autopsy, and diagnosis largely relies on parasitologic and histopathologic examination (*2**,**14**,**15*). Recently, PCRs have been developed for canine pentastomiasis (*9*), but DNA sequences in the databases are limited to a few species of pentastomes only.

We have provided partial 18S rRNA gene sequences of *A. armillatus* pentastomes and used PCR for the diagnosis of pentastomiasis from a clinical sample (*9*). We also constructed phylogenetic trees for all pentastome species infecting humans and animals from which sequence data were available. Phylogenetic analysis showed that pentastomes formed their own branch in proximity to the Branchiura and Remipedia, a finding which is consistent with results of a previous study by others who investigated *A. armillatus* as a sole member of the pentastomes in comparison with pancrustaceans (*1*). The nearest phylogenetic relatives of *A. armillatus* are *A. agkistrodontis* and *P. crotali*, 2 species of the Porocephalida, followed by *L. serrata* and by the members of the Cephalobaenida (pentastomes that infect birds). The phylogenetic trees constructed here indicate a coevolution of the pentastomes and other maxillopodan/branchiuran parasites with their vertebrate hosts (birds, snakes, mammals, and fish).

We demonstrated that the serum of a patient with linguatuliasis markedly cross-reacted on the ELISA and Western blot based on *Armillifer* spp. antigens. To prevent further infections, personal hygiene measures were implemented, such as thorough hand cleansing after handling snakes and avoidance of contact with snake excretions. Public health institutions have been informed, and future studies will address the extent of seroprevalence in the local population.
